# Formulation of *Entandrophragma utile* into an Herbal Emulgel for the Management of Inflammation

**DOI:** 10.3390/gels9120956

**Published:** 2023-12-06

**Authors:** Adeola Tawakalitu Kola-Mustapha, Haneefat Folashade Ibraheem, Suleiman Taiwo, Ismail O. Ishola, Sukurat Olasumbo Usman, Yusuf Oluwagbenga Ghazali

**Affiliations:** 1 College of Pharmacy, Alfaisal University, Riyadh 11533, Saudi Arabia; 2 Department of Pharmaceutics and Industrial Pharmacy, Faculty of Pharmaceutical Sciences, University of Ilorin, Ilorin 240101, Nigeria; 3 Department of Pharmacology, Therapeutics and Toxicology, Faculty of Basic Medical Sciences, College of Medicine, University of Lagos, Lagos 100001, Nigeria; 4 Department of Pharmacognosy and Drug Development, Faculty of Pharmaceutical Sciences, University of Ilorin, Ilorin 240101, Nigeria

**Keywords:** inflammation, herbal emulgel, *Entandrophragma utile*

## Abstract

**Introduction:** Globally, the incidence of inflammation and inflammatory disorders has continued to rise at an alarming rate. *Entandrophragma utile* is a species of flowering plant widely distributed in Africa and has been used for the management of sickle cell disease, rheumatism, ocular inflammation, duodenal and stomach ulcers. This research aims to formulate and evaluate an anti-inflammatory herbal emulgel using an extract from *Entandrophragma utile* stem bark (EUB). **Method:** Using a carrageenan-induced paw oedema model, the anti-inflammatory efficacy of EUB the extract was assessed. The formulated *Entandrophragma utile* emulgels (EUE) were characterized, and their anti-inflammatory activity was demonstrated, by utilizing diclofenac emulgel-treated rats with complete Freund’s adjuvant (CFA)-induced arthritis model as the positive control group. **Results:** The emulgels formulated had characterization results within acceptable ranges; pH (4.25–5.80), viscosity (418.9–112.8 mPas), spreadability (25.00–31.82 gcm/s), extrudability (30.86–51.02 g/cm^2^), and a swelling index of (30–60%). The emulgel produced a concentration-dependent inflammatory inhibition with a peak effect (117.97%) at the end of the 4th week which was comparable to that of commercial diclofenac (127.19%). The phytochemical analysis led to the identification of saponins, flavonoids, phenols, and tannins as active secondary metabolites. **Conclusions:** The stem bark extract of *E. utile* possessed noteworthy (*p* < 0.05) reduction in inflammation in comparison to diclofenac and its emulgel formulation showed enormous potential for treating inflammation and pain.

## 1. Introduction

Globally, inflammation is a major issue because of its crippling signs, leading to the loss of productivity and significant suffering [1]. Inflammation, derived from the Latin word ‘*inflammatio*’, is a defence mechanism of the body against diseases or undesired external substances that infiltrate tissue cells [2]; with five vital indications- redness, swelling, heat, pain, and loss of function [3]. There are a variety of chemical mediators (which are either cell or plasma-derived) from the circulatory damaged tissue, inflammatory cells, and the immune system that actively participates in, and adjusts inflammatory responses [4]. They include vasoactive amines such as histamine and serotonin, peptides such as bradykinin; and eicosanoids such as thromboxane, leukotrienes, and prostaglandins. Inflammation could be acute [5] or chronic [6].

Acute inflammation occurs when there is tissue damage as a result of injury, microbial invasion or toxic compounds. It begins rapidly and becomes severe within a little duration of time and symptoms may persist for a few days, such as in the case of cellulitis or acute pneumonia [7]. When acute inflammation becomes non-regulated, it may advance to chronic inflammation. Chronic inflammation is a protracted inflammation that lasts for longer than a few months or a few years. The reason for the injury and the body’s capacity to heal and overcome the damage are the main determinants of the degree and consequences of chronic inflammation. [7]. It is essential for the emergence of several pathological conditions, including cancer, rheumatoid arthritis, obesity, diabetes type 2, Alzheimer’s disease, along with pulmonary and cardiovascular diseases [8].

The leading cause of death globally has been associated with chronic disorders [9], with 60% of these deaths due to chronic inflammatory diseases [7]. Although, there are several classes of drugs currently available for the treatment of inflammation, non-steroidal anti-inflammatory medications (NSAIDs) continue to be the most widely utilized treatment for these ailments. Unfortunately, the clinical use NSAIDs is often associated with some undesirable side effects [7] and hence, the need for the search for alternative therapies are essential. NSAIDs provide pain relief and anti-inflammatory activity of great value and therefore, the goal is not to replace NSAIDs altogether, but to decrease the frequency of dosages. According to WHO, it is estimated that in developing countries, about 80% of the population uses traditional medicines for their health care [10]. For thousands of years, medicinal plants have been utilized as traditional remedies for a wide range of human ailments in many different parts of the world. In rural areas, medicinal plants are still the primary source of medicine since they have anti-inflammatory properties with minimal to no adverse effects. [11].

*Entandrophragma utile* is a species of flowering plant in the Meliaceae family known by the local names- Igi jebo (Yoruba); Sipo, Assie, Acajou (French); Mukola, Muyovu (Luganda) [12]. The plant is widely distributed in the Guinea-Congolese region, particularly growing in the evergreens and semi-deciduous dense rainforests. It is found in large areas that stretches both north and south of the equator, extending from the Atlantic coast across the Congo basin to the eastern side of the Kivin Ridge. It may also be found in Angola, Cameroon, Central African Republic, Congo DR, Cote d’Ivoire, Gabon, Ghana, Liberia, Nigeria, Sierra Leone, and South of the African Sahara [13]. In Nigeria, people have long utilized the stem bark of *E. utile* to cure conditions like rheumatism, ocular irritation, sickle cell illness, stomach, and duodenal ulcers, and more [14,15]. Phytochemical analysis of this plant has led to the identification of secondary metabolites such as saponins, flavonoids, phenols, and tannins, with alkaloids and phytosterols both absent [16]. 

Despite reports of the traditional use of *E. utile* to treat gastrointestinal diseases, sickle cell anaemia, and rheumatism in local communities across Africa, only a few studies have been able to corroborate these claims using standard pharmacological experiments. Unlike some other Meliaceae species like *Azadirachta indica* that have been well-studied for their therapeutic activity against inflammation [17], there is still a dearth of data on the pharmacological application of *E. utile* in the management of inflammation. This is the knowledge gap this research project seeks to fill. In addition to deploying an acceptable pharmacological model to establish the anti-inflammatory activity of *E. utile* bark extract in rats, this research is particularly novel in its drug presentation approach. It constitutes the first attempt at formulating the stem bark extract of *E. utile* into a versatile dosage form like an emulgel and delivering it for potential topical administration. 

Drugs are designed in different ways to effectively deliver the active ingredients to their specific sites of action for the exertion of therapeutic action. Topical formulations which are directly applied to the skin have the advantage of bypassing first-pass metabolism, enhancing patient compliance also, preventing gastrointestinal incompatibility [18]. Emulgels, a class of topical dosage forms, are formed when gels and emulsions are combined and their thixotropic, greaseless, easily spreadable, readily removable, emollient, non-staining, water-soluble, longer shelf life, bio-friendly, translucent, and aesthetically pleasant nature are just a few of the advantageous qualities for dermatological use. In this research, the anti-inflammatory activity of *Entandrophragma utile* will be studied towards the formulation of an herbal-based topical dosage form which can potentially become a suitable alternative to the use of NSAIDs in the management of pain and inflammation.

## 2. Results and Discussion

### 2.1. Percentage Yield

The percentage yield of the ethanol extract of powdered stem bark of *E. utile* was 9.0% as calculated below:

$$=\frac{72.07\;g}{800\;g}\times 100$$

=9.0% (ww)

### 2.2. Anti-Inflammatory Assay of the Extract

The extract’s anti-inflammatory assay was measured by a decrease in the rats’ paw size (mm) over 24 h; results are presented in Table 1 below.

### 2.3. Emulgel Characterization

Table 2 displays the physical characteristics of the emulgels that were formulated, such as their appearance, color, odor, homogeneity, and ease of application and removal.

### 2.4. Extrudability, Spreadability, pH, and Swelling Indices of Formulated Emulgels

In terms of the weight of the load divided by the area of the ribbon that was extruded, the extrudability of the emulgels made from an aluminum foil tube ranged from 30.86 to 51.02 g/cm^2^ (Table 3). The emulgels that were prepared according to Table 3 had a spreadability of 25.00–31.82 gcm/s. The emulgels were tested for pH, and the results ranged from 4.25 to 5.80 (Table 3). Furthermore, the emulgels’ swelling indices varied from 30 to 60%. (Table 3).

### 2.5. Viscosity

The results obtained from the determination of the viscosity attributes of the formulated emulgel are presented in Table 4.

### 2.6. Skin Irritation and Anti-Inflammatory Assay of Emulgels

**Figure 1:**
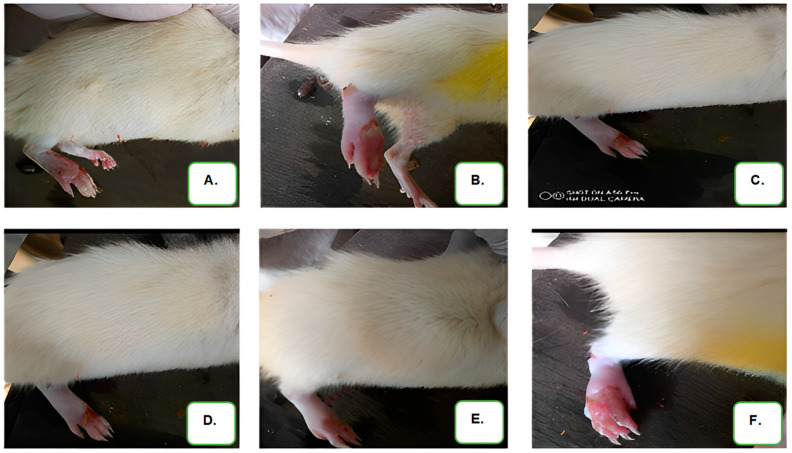
Image showing the effect of the treatments (with the formulated herbal emulgel) on CFA-induced inflammation in the rats. [(**A**)-EUE1, (**B**)-EUE2, (**C**)-EUE3, (**D**)-EUE4, (**E**)-EUE5, (**F**)-DIC].

The outcomes showed that the prepared emulgels were free from dermatological reaction and did not cause irritation, redness, or edema upon application. Results of the anti-inflammatory assay performed on the emulgel are presented in Table 5 below. In addition to this, Figure 1 shows the effects of the formulated emulgel on CFA-induced inflammation in the test rats. 

It has been shown that different nonsteroidal anti-inflammatory medications lessen inflammation and pain through the blockage of arachidonic acid metabolism by isoforms of cyclooxygenase enzyme, which leads to reduced prostaglandin production. Regrettably, using nonsteroidal anti-inflammatory medications can have several negative effects. It is interesting to note that a variety of medicinal plants have anti-inflammatory therapeutic benefits. These medicinal plants also have little to no adverse effects [19]. Medicinal plants have been the chief remedy in the treatment of various type of diseases and ailments in a long period of time; and more recently, many drugs have been developed from traditional medicine [20]. 

The stembark of *E. utile* has been traditionally used for the management of sickle cell illness, rheumatism, duodenal and stomach ulcers, and eye irritation in Nigeria. Saponins, flavonoids, phenols, and tannins are examples of secondary metabolites that have been identified [16]; and the pharmacological potency observed in medicinal plants is due to the presence of these bio-active secondary metabolites [16]. The percentage yield of the plant extract (calculated to be 9.0%(*w*/*w*)) was a similar yield to what was obtained by Usman et al., 2018 [16]. To make sure the anti-inflammatory activity of the *E. utile* extract was determined via a mechanism appropriate for the application of an experimental plant part, the anti-inflammatory test was performed using the carrageenan-induced rat paw model. Non-steroidal anti-inflammatory medications are also studied using the rat paw model caused by carrageenan [21]. The inhibitory properties of the plant extracts were examined at different concentrations as shown in Table 1, and it was observed that 100 mg/kg and 200 mg/kg had percentage (%) inhibition of 43.62 and 15.96% respectively, which was significantly (*p* < 0.05) more than diclofenac, a non-steroidal anti-inflammatory drug. This formed the basis of the formulation of a topical preparation with the extract grading from 0.5–2%.

The presence of phytochemicals in plant parts is the reason many herbs and their derivatives show useful therapeutic advantages when used either in their crude forms or as formulated pharmaceutical dosage forms. Like in other plants belonging to the Meliaceae family, the presence of the secondary metabolite–limonoids, is primarily responsible for their pharmacological actions [22]. This secondary metabolite (limonoids) is present in the stem back of *E. utile* [23] and can be said to be involved in the anti-inflammatory activity elicited by the extract in the test rats used in this study. Furthermore, Hu et al. (2020) in their research found eight new compounds (comprising of four limonoids, two steroids, one triterpenoid and one lignan) in their phytochemical analysis of the stem bark of *E. utile* [24]. Since these isolated phytochemicals were reported to have also shown potent anti-inflammatory effect, it is not out of place to attribute the anti-inflammatory activities displayed in this research to the presence of the reported phytochemicals. Developing a topical dosage form (like an emulgel) from the bark extract of *E. utile* is therefore imperative, to ensure that its constituent phytochemicals are suitably delivered for optimum therapeutic action. 

An emulgel is an intriguing topical medication delivery system with gel and emulsion dual release systems. It has significant potential to function as a drug delivery vehicle by combining a variety of medicinal molecules and has several other favorable properties for dermatological use. Therefore, it can be used as better topical drug delivery system over present systems [25,26]. During the formulation process, a preliminary analysis was carried out on different mixing ratios of Carbopol 940 to Ultrez Carbopol to give the best results when combined, and 30:70 gave the best characteristic gel base. The emulgels formulated based on this had satisfactory physical characteristics. All the emulgels had minty smell with the intensity of the odor decreasing with increased concentration of the extract, giving E1 (the control) the most minty odor and E5 with 2% extract a characteristic ‘bark’ smell. The different shades of brown (from light-caramel-deep brown) observed in the formulated emulgels can be attributed to the bark extract which is deep brown in color. The pH values of the formulations (Table 3) were found to be slightly acidic with an observed increment with increasing concentration of extract. This observation can be explained to be a consequence of the high value of tannins (an acidic compound), present in the extract [16,27]. With reference to other reports from literature, there is a high level of agreement that topical products should be acidic and should possess pH in the range of 4 to 6 [28]. 

Spreadability is an important characteristic of semi-solid formulations and is responsible for the ease of application on the substrate, extrudability from the package, correct dosage transfer to the site of action, and patient compliance [29]. Additionally, the efficacy of a topical dosage form is dependent on the patient spreading the drug formulation in an even layer to administer a standard dose. All the emulgel formulations were of adequate spreadability and the values obtained (Table 3) indicated that emulgels are easily spreadable when small amount of shear is applied with an ability to cling to the surface for a while pending the time they are worn or washed off. The viscosity of all the formulations were measured at 12 rpm (E1–E4) and 30 rpm (E5) at the same temperature. The formulations showed decrease viscosity with increase shear rate of 12 to 30 rpm, with E5 at 30 rpm been the least viscous with 112.8 mPas reading. This is because steep decrease in viscosity will occur as solid fractions decreases and shear rate increases [30]. The extrudability of the herbal emulgel formulations fell in between 25.00 and 51.0 g/cm^2^ range, indicating good extrudability. Quantifying extrudability is important to determine the ease of removal and application of products as well as patient acceptance [31]. The emulgels had different swelling index values (30–55%), this variation may be dependent on the water uptake nature and chain strength of the polymers used as gelling agent-Carbopol 940 [32]. Swelling to a larger size extend systemic retention [33].

The effect of the formulated emulgels on chronic inflammation was examined in an arthritic model produced by complete Freund’s adjuvant (CFA). When CFA was injected into the rats’ hind paws, there was a noticeable increase in joint inflammation. This inflammation was caused by activated macrophages and the release of several enzymes into the bloodstream, which over time contributed significantly to tissue degradation, fibrosis, and vascular damage. [34]. Treatment of rat with the formulated emulgels resulted in a dose-dependent effect. The emulgels showed good results as compared to diclofenac emulgel across the test days with few exceptions. E5 produced the highest % inhibition at the end of the 4th week (117.97%) which was comparable to the standard diclofenac gel with % inhibition of (127.19%). This indicates that E5 with 2% extract reduced inflammation considerably by the end of the 4th week. The results obtained from the emulgel characterization and anti-inflammatory models using carrageenan revealed that emulgel containing *Entandrophragma utile* extract functions well as a topical delivery method in the management of pain and inflammation.

## 3. Conclusions

The results of this investigation demonstrate the anti-inflammatory properties of *E. utile* stem bark extract. This justifies its use in the preparation of the herbal anti-inflammatory emulgel that was effectively created using the extract as the active ingredient in various quantities. The emulgels upon evaluation were found to be mostly satisfactory. The extract formulated into emulgel formulation therefore has a lot of potential for treating pain and inflammation.

## 4. Materials and Method

### 4.1. Chemicals and Equipment

Ethanol (BDH Chemicals Ltd., Dorset, England), Tween 80 (BDH Chemicals Ltd., Dorset, England), Span 20 (BDH Chemicals Ltd., Dorset, England), Methylparaben (BDH Chemicals Ltd., Dorset, England), Ethylparaben (BDH Chemicals Ltd., Dorset, England), Carbopol 940 (Guangdong Weng Jiang Chemical Reagent Co. Ltd., Guangdong, China), Ultrez Carbopol 20 (BDH Chemicals Ltd., Dorset, England), Liquid paraffin (BDH Chemicals Ltd., Dorset, England), Propylene glycol (BDH Chemicals Ltd., Dorset, England), triethanolamine (BDH Chemicals Ltd., Dorset, England), Clove oil (Ashifaul-Haqi Ltd., Oyo, Nigeria), Mentha oil (Ashifaul-Haqi Ltd., Oyo, Nigeria), Distilled water (Department of Pharmaceutical Chemistry, University of Ilorin, Kwara, Nigeria), Water bath (Fisher Scientific Company, Pittsburgh, PA, USA), Analytical weighing balance (Ohaus, Parsippany, NJ, USA), pH meter (Hanna Instruments Ltd., Bedfordshire, England),Viscometer (NDJ-5S, Rinch Industrial Co. Limited, Shanghai, China). 

### 4.2. Collection and Identification of Plant

Fresh stem barks of *Entandrophragma utile* were gathered at Olokemeje, Oyo State (Nigeria). They were identified and verified at the Department of Plant Biology Herbarium Unit, University of Ilorin, Nigeria, in which voucher specimen (UILH/001/1325) was logged in.

### 4.3. Preparation of Extract

The stem barks were dried and ground using a mechanical grinder. The powdered plant materials were exhaustively extracted by maceration for 72 h using 90% *v*/*v* ethanol at room temperature with occasional stirring [35], after which it was filtered and concentrated using a rotary evaporator. The dried extract which was reddish brown in colour was stored for later use. The percentage yield of the extract was determined using the formula:

$$\mathrm{Percentage}\;\mathrm{yield}=\frac{weight\;of\;solid\;extract\;obtained}{weight\;of\;powdered\;stem\;bark\;used}\times 100\%$$

### 4.4. Characterization of Extract 

The extract of *Entandrophragma utile* was assessed for physical attributes like color, feel, and scent. 

### 4.5. Anti-Inflammatory Assay of Extract

Every experiment was conducted in compliance with the recommendations set by University’s Ethical Review Committee (UERC). Using animal models, the anti-inflammatory assay was conducted. For the anti-inflammatory investigation, albino Wistar rats were allowed to acclimatize to the laboratory environment for seven days and were fasted for 18 h prior to the trial.

#### Carrageenan-Induced Rat Paw Edema Method

Five groups of twenty (20) rats each were equally divided. Four test groups received oral pretreatment with plant extract of dose levels of 50, 100, 200 mg/kg and diclofenac respectively an hour before Carrageenan (0.1 mL of 1% suspension in normal saline) was injected sub-plantarly into the rats’ right hind paws to produce acute inflammation. The measurements of the paw circumference were taken 24 h following the injection of carrageenan. The volume of oedema and the percentage of anti-inflammatory activity were computed using the difference between the readings [36]. Using the following formula, the percentage value of oedema inhibition was determined:

Inhibition percentage = 1 − (y − x/b − a) × 100

where a = Initial paw thickness of the test group animal; b = Paw thickness of the control group animal following treatment; and x = Initial paw thickness of the test group animal [37].

### 4.6. Emulgel Formulation

The emulgel was made using Mohamed’s (2004) methodology with a little modification [38]. To create the gel bases, dispersing a weighed and sieved quantity of Carbopol 940 in a measured quantity of distilled utilizing a mechanical shaker to stir continuously at a reasonable speed, and letting it soak for the entire night. The Ultrez Carbopol 20 was dispersed in a measured quantity of distilled water and constantly stirred using mechanical shaker until gel formed. Both gels were mixed and properly stirred until a homogenous mixture was obtained. The pH of the gel was tested and adjusted by adding drops of triethanolamine. The emulsion’s aqueous phase was created by dissolving Tween 80 in filtered water, while the oil phase was created by dissolving Span 80 in light liquid paraffin. Propylene glycol was used to dissolve methyl and propylparaben before being combined with the aqueous phase. Because it was hydrophilic, the plant extract dissolved in the aqueous phase. The aqueous and oily phases were heated to 70–80 °C individually. Then, the oily phase was introduced to the aqueous phase and stirred continuously until it cooled to room temperature. To create the emulgel, the emulsion was combined with the gel in a 1:1 ratio while being gently stirred. Table 6 shows the composition of several formulations.

### 4.7. Characterization of Emulgels

#### 4.7.1. Physical Appearance

Visual inspection was conducted to assess the pH, color, homogeneity, consistency, grittiness, and phase separation of the manufactured emulgel formulations.

#### 4.7.2. Measurement of pH

A digital pH meter (Hanna, England) was used to measure the pH of the emulgel formulations. The probe was left in the test preparation for two hours after one gram of gel was dissolved in 100 millilitres of distilled water. Each formulation’s pH was measured three times, and the average results were determined.

#### 4.7.3. Spreadability

The diameter of the emulgel circle formed when emulgels are sandwiched between two glass plates of a specific weight were used to gauge how spreadable emulgels are. One glass plate was used to weigh an amount of each emulgel, while another glass plate was dropped from seven centimetres. The diameter of the emulgel spread’s circle was measured.

#### 4.7.4. Viscosity

Utilizing an NDJ-5S viscometer (Spindle type no. 3) at 12 and 30 rpm, the estimation of viscosity was carried out. A significant amount of emulgel was placed in a beaker, and the spindle was plunged into it for five minutes before readings were obtained.

#### 4.7.5. Extrudability

The extrudability test measures the weight needed to extrude a 0.5 cm emulgel ribbon from a lacquered collapsible aluminum tube in 10 s. The test was run three times, and the average values were determined. The following formula was then used to determine the extrudability:

Extrudability = Weight used to extrude emulgel from a tube (in g)/area (in cm^2^).

#### 4.7.6. Swelling Index

This is accomplished by adding 1 g of the emulgel to a 25 mL stoppered cylinder, adding water to reach the 20 mL mark, gently shaking the cylinder every 24 h, and letting it stand. The weights of the swollen mass were then calculated.

[(Wt − Wo)/Wo] × 100 is the swelling index (SW) percentage.

where % {SW} = Perfect Swelling Equilibrium.

Wt = the weight of the swelled emulgel after time t,
Wo = the emulgels initial weight at time zero.

#### 4.7.7. Skin Irritation Test 

The formulations were rubbed on the back of the palm and observed for any irritation.

### 4.8. Anti-Inflammatory Assay of Emulgels 

#### CFA-Induced Arthritic Model

Experimental rats were divided into six (6) groups (Group 1–6), and they were treated with the formulated emulgels (EUE1–EUE5) and diclofenac (positive standard) respectively. EUE1 containing 0% of extract served as the negative control. Each animal’s paw volume at 0 days was measured before the experiment. Then, each rat’s right hind paw’s subplantar tissue was subcutaneously injected with 0.1 mL of CFA to cause adjuvant arthritis. For 27 days, the circumference of each paw was measured every day with a vernier caliper. 

Using the following formula, the percentage value of oedema inhibition was determined:

Inhibition percentage = 1 − (y − x/b − a) × 100

where a = Initial paw thickness of the test group animal; b = Paw thickness of the control group animal following treatment; and x = Initial paw thickness of the test group animal. [37,39].

### 4.9. Statistical Analysis

Mean ± SEM was used to present the obtained results. For the statistical comparison, one-way analysis of variance (ANOVA) was employed, followed by the Dunnett post hoc multiple comparison tests for the anti-inflammatory examination. Graph-Pad Prism version 6 (GraphPad Software, Inc., San Diego, CA, USA) was used for statistical analysis. A significance threshold of *p* < 0.05 was used for analysis. 

## Figures and Tables

**Table 1:** Extract’s anti-inflammatory assay measured by a decrease in the rats’ paw size (mm).

Treatments	Mean Change in Paw Size (mm)					
	0 h	1 h	2 h	3 h	4 h	24 h
Control	1.22	1.57	1.57	1.67	1.62	1.53
EUB (50 mg/kg)	1.21	1.98	1.81	1.87	1.77	1.50
**%** inhibition		−17.92	−68.87	−45.93	−39.17	−7.44
EUB (100 mg/kg)	−1.22	1.80	1.82	1.86	1.73	1.40
**%** inhibition		−64.16	−70.75	−41.48	−28.33	43.62 *
EUB (200 mg/kg)	1.21	1.80	1.80	1.79	1.65	1.48
**%** inhibition		−66.03	−66.98	−27.41	−8.33	15.96 *
Diclofenac	1.24	1.76	1.72	1.76	1.69	1.53
**%** inhibition		−48.11	−36.79	−14.81	−12.50	−4.25

Data presented as Mean ± SEM (*n* = 3), * Indicates significant inhibition.

**Table 2:** Physical characterization of the *Entandrophragma utile* herbal emulgels.

Emulgel	Appearance	Color	Odor	Ease of Application	Ease of Removal	Homogeneity	Phase Separation
**EUE** **1**	Viscous, creamy	White	Minty odour	Very easily applied	Easily removed	Homogenous	No
**EUE** **2**	Viscous, creamy, glossy	Light brown	Minty with slight bark odour	Very easily applied	Easily removed	Homogenous	No
**EUE** **3**	Viscous, creamy, glossy	Light brown	Minty with bark odour	Very easy applied	Easily removed	Homogenous	No
**EUE** **4**	Viscous, creamy, glossy	Caramel brown	Minty with bark odour	Very easy applied	Easily removed	Homogenous	No
**EUE** **5**	Viscous, creamy, glossy	Deep brown	Minty with characteristic bark odour	Very easy applied	Easily removed	Homogenous	No

**Table 3:** Some evaluation parameters for *Entandrophragma utile* herbal emulgels.

Emulgels	pH	Spreadability (gcm/s)	Extrudability (g/cm^2^)	Swelling Index %
**EUE** **1**	4.25	30.43	51.02	30.00
**EUE** **2**	4.57	31.82	39.06	50.00
**EUE** **3**	4.60	26.92	25.00	60.00
**EUE** **4**	4.76	24.14	39.06	30.00
**EUE** **5**	5.80	25.00	30.86	55.00

**Table 4:** Viscosity of the formulated emulgels taken at 12 and 30 rpm.

Emulgels	°C	SPL	RPM	%	Readings (mPas)
**EUE1**	33.6	1	12	83.8	418.9
**EUE2**	33.1	1	12	52.6	262.8
**EUE3**	33.8	1	12	72.4	362.1
**EUE4**	32.3	1	12	51.8	259.1
**EUE5**	34.5	1	30	56.4	112.8

**Table 5:** Anti-inflammatory assay of the emulgel as reduction in paw size of rats (mm) for 4 weeks.

Formulation	Mean Change in Paw Size (mm)								
	Day 3	Day 6	Day 9	Day 12	Day 15	Day 18	Day 21	Day 24	Day 27
**EUE1**	3.029 ± 0.18	3.663 ± 0.08	4.285 ± 0.18	4.905 ± 0.29 *	5.061 ± 0.4 *	5.204 ± 0.38 *	4.996 ± 0.36 *	4.525 ± 0.34 *	3.924 ± 0.36 *
**EUE2**	3.006 ± 0.21	3.863 ± 0.24 *	3.766 ± 0.26	3.668 ± 0.29	3.636 ± 0.33	3.439 ± 0.32	3.374 ± 0.38	3.01 ± 0.32 *	2.503 ± 0.28 *
**% inhibition**	5.09	−8.84	22.69	41.48	45.29	53.45	52.45	57.65	69.71
**EUE3**	2.885 ± 0.16	3.774 ± 0.42 *	3.538 ± 0.32	3.606 ± 0.27	3.441 ± 0.31	3.096 ± 0.23	2.889 ± 0.17	2.268 ± 0.23	1.781 ± 0.20
**% inhibition**	14.86	−4.2	31.83	43.36	51.21	63.5	67.65	85.04	104.01
**EUE4**	2.979 ± 0.19	3.471 ± 0.29	3.518 ± 0.21	3.798 ± 0.22	3.888 ± 0.24 *	3.419 ± 0.27	2.831 ± 0.27	2.29 ± 0.22	1.861 ± 0.24
**% inhibition**	8.93	13.59	33.58	37.87	38.08	54.61	70.21	85.08	101.29
**EUE5**	3.684 ± 0.17 *	4.121 ± 0.22 *	4.043 ± 0.2	3.608 ± 0.35	4.081 ± 0.15 *	3.754 ± 0.16 *	3.133 ± 0.21	2.314 ± 0.14	1.538 ± 0.08
**% inhibition**	−47.75	−20.47	13.24	44.89	32.91	45.45	61.49	85.15	117.97
**DIC**	2.891 ± 0.11	3.38 ± 0.24	3.115 ± 0.21	2.911 ± 0.17	2.748 ± 0.19	2.606 ± 0.16	2.46 ± 0.13	1.767 ± 0.05	1.279 ± 0.03
**% inhibition**	12.94	16.38	48.37	65.42	72.14	77.53	80.66	103.01	127.19

Values are expressed as mean ± SEM (n = 8). The vehicle-treated control was compared to the formulations by one-way ANOVA followed by Dunnett post hoc multiple comparison test setting statistically significant level at (*p* < 0.05). * Indicates mean with statistical difference between formulation and Diclofenac.

**Table 6:** Composition of *Entandrophragma utile* emulgel formulations (%*w*/*w*).

Ingredients	Composition (%*w*/*w*)				
	EUE1	EUE2	EUE3	EUE4	EUE5
Extract	0.00	0.50	0.10	1.50	2.00
Carbopol-940	0.30	0.30	0.30	0.30	0.30
Alcohol	2.50	2.50	2.50	2.50	2.50
Ultrez Carbopol	0.70	0.70	0.70	0.70	0.70
Span 20	1.00	1.00	1.00	1.00	1.00
Tween 80	0.50	0.50	0.50	0.50	0.50
Liquid Paraffin	7.50	7.50	7.50	7.50	7.50
Methyl Paraben	0.03	0.03	0.03	0.03	0.03
Propyl Paraben	0.01	0.01	0.01	0.01	0.01
Peppermint Oil	6.00	6.00	6.00	6.00	6.00
Propylene Glycol	5.00	5.00	5.00	5.00	5.00
Water	q. s	q. s	q. s	q. s	q. s

## Data Availability

The data presented in this study are openly available in the article.
